# Harnessing the potential of *Lactobacillus* species for therapeutic delivery at the lumenal-mucosal interface

**DOI:** 10.2144/fsoa-2020-0153

**Published:** 2021-02-04

**Authors:** Joseph R Spangler, Julie C Caruana, Igor L Medintz, Scott A Walper

**Affiliations:** 1 National Research Council Postdoctoral Fellow sited in US Naval Research Laboratory, Code 6900, Center for Bio/Molecular Science & Engineering, 4555 Overlook Ave SW, Washington DC, 20375, USA.; 2 American Society for Engineering Education Postdoctoral Fellow sited in US Naval Research Laboratory, Code 6900, Center for Bio/Molecular Science & Engineering, 4555 Overlook Ave SW, Washington DC, 20375, USA.; 3 US Naval Research Laboratory, Code 6900, Center for Bio/Molecular Science & Engineering, 4555 Overlook Ave SW, Washington DC, 20375, USA

**Keywords:** cytokine stimulation, delivery vehicle, immunomodulation, *Lactobacillus*, microbiome, probiotic, therapy, vaccine

## Abstract

*Lactobacillus* species have been studied for over 30 years in their role as commensal organisms in the human gut. Recently there has been a surge of interest in their abilities to natively and recombinantly stimulate immune activities, and studies have identified strains and novel molecules that convey particular advantages for applications as both immune adjuvants and immunomodulators. In this review, we discuss the recent advances in *Lactobacillus*-related activity at the gut/microbiota interface, the efforts to probe the boundaries of the direct and indirect therapeutic potential of these bacteria, and highlight the continued interest in harnessing the native capacity for the production of biogenic compounds shown to influence nervous system activity. Taken together, these aspects underscore *Lactobacillus* species as versatile therapeutic delivery vehicles capable of effector production at the lumenal-mucosal interface, and further establish a foundation of efficacy upon which future engineered strains can expand.

## Lay abstract

Bacteria such as *Lactobacillus* species have traditionally maintained positive association with gastrointestinal health. This review summarizes recent research relating to human health associated *Lactobacillus* administration on cellular and systematic levels. As techniques in molecular and synthetic biology continue to advance, the potential to increase the therapeutic scope and efficacy of *Lactobacillus* cultures increases regarding target molecule production, immune system influence, vaccination and nervous system activities.

The rising incidence of systemic diseases is one of the many driving factors for therapeutic development [1,2]. The effort to develop, test and market treatment options for a vast number of health-related issues is a difficult undertaking. Companies within the US alone invest billions annually in research and development of new therapeutics and treatments [3]. The need for cellular targeting and specific therapeutic delivery has grown with prevalence of indiscriminate cancer treatment techniques and their associated off-target effects, and this has led to the development of many fascinating technologies in past decades such as decorated nanoparticles, for example [4]. A delivery system of particular interest, and the focus of this review is one stemming from resident gut commensal bacteria. The gut microbiome represents a complex and diverse population of bacteria that maintain a versatile range of metabolic capabilities, and as such they affect host systems across a spectrum of beneficial and deleterious ends.

The composition of the microbiome is variable and highly elusive, but certain bacterial groups have arisen through study in past decades to be recognized for their probiotic characteristics, wherein health benefits observed in human populations can be correlated to the inclusion of these bacteria in their customary diets [5]. As a result, a large number of investigators have dedicated time to isolating these bacteria directly from traditional food sources and testing them *in vitro* and *in vivo* in an effort to elucidate the host-beneficial mechanisms [6–14]. Many of these bacteria belong to the genus *Lactobacillus*, a collection of Gram-positive bacteria many of which are generally regarded as safe food-grade organisms commonly found as commensals in the animal gut microbiota. These bacteria have been studied for both their industrial applications in the production of flavor compounds and other fermentative abilities [15], in addition to their abilities to alleviate medically elusive systemic issues in the large intestine involving inflammation [16]. *Lactobacillus* belongs to the larger lactic acid bacteria (LAB) group, for which genetic tools have been developed in efforts to increase their potential as immune adjuvants and cell factories charged with the secretion of bioactive molecules [17]. In effect, these microbes can be administered orally, live in the gut and deliver therapeutic benefits as well as targeted compounds through their interactions with the host immune and nervous systems (Figure 1). In this review, we discuss the *Lactobacillus*-related activity at the gut-microbiota interface such as the immunomodulating effects of these bacteria on immune and epithelial cells. We further highlight the discovery efforts for native compounds produced by these bacteria that can be delivered directly to the epithelia for immunomodulation, as well as the use of recombinant techniques to produce living vaccines to stimulate adaptive immunity. Finally, we discuss recent insights into the role of these bacteria in the production of and influence on native biogenic compounds capable of stimulating the nervous system in order to emphasize the direct and indirect therapeutic potential of these bacteria for the treatment of a diverse set of diseases.

**Figure 1.**
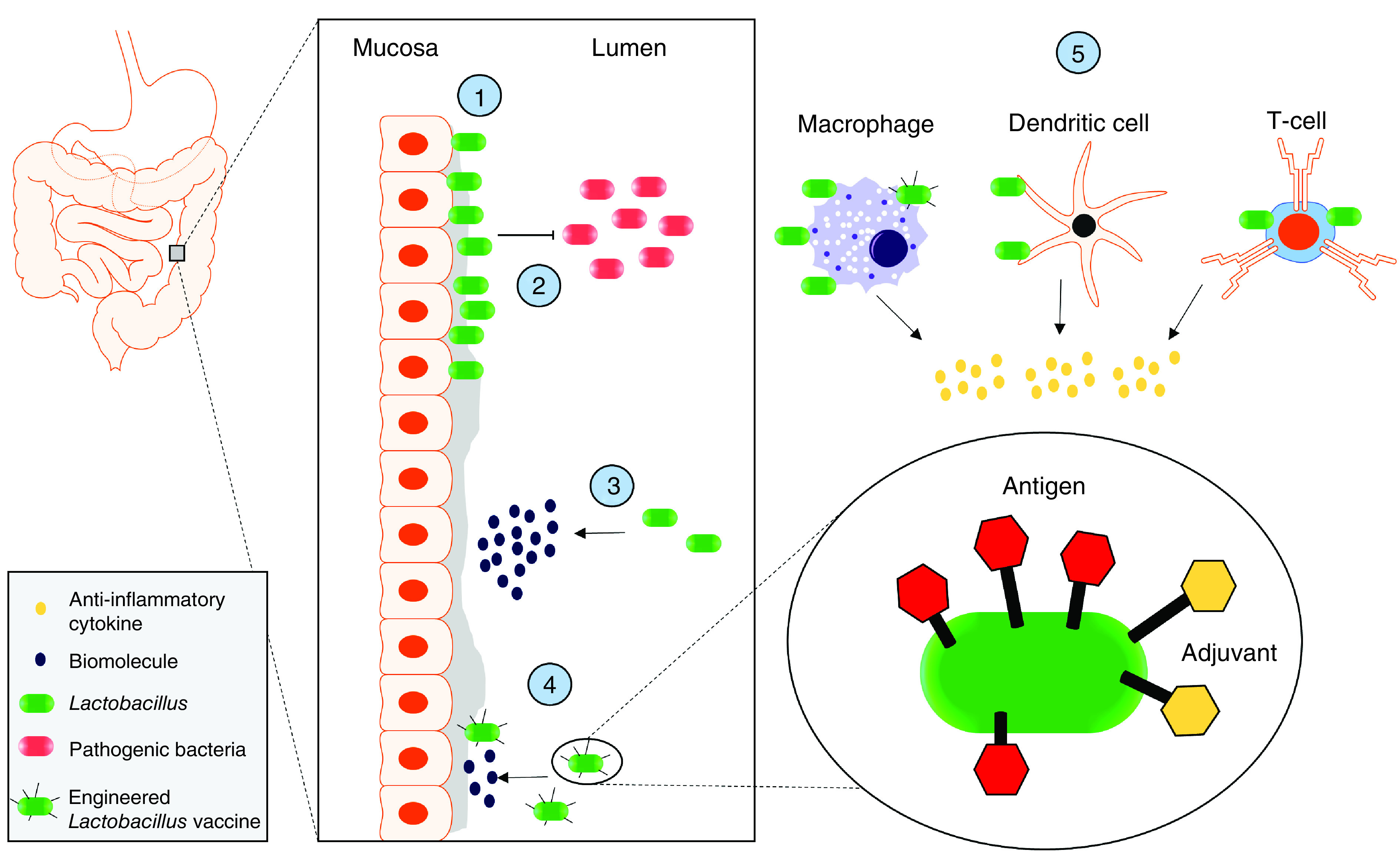
Overview illustrating interactions between *Lactobacillus* and host discussed in this review. **(1)** Adhesion of *Lactobacillus* cultures to the epithelial barrier at the Lumen–Mucosa interface within the large intestine, **(2)** the prevention of pathogen adhesion to that barrier through physical exclusion that prevents pathogen invasion and virulence, **(3)** the production and secretion of recombinantly and natively produced biomolecules for uptake/recognition by host cells, **(4)** the display of antigens and adjuvants through various linking strategies for localized vaccine delivery to the mucosa and **(5)** the interactions with immune cells resulting in their secretion of anti-inflammatory cytokines.

## Interactions with host cells

### Influence on immune cell behavior

The probiotic properties of LAB have been studied for decades [18]. The study of their beneficial characteristics has mainly centered on general physiological effects such as decreased susceptibility to infection [19], but in recent years there has been a considerable effort to isolate and study specific bacteria to better understand the molecular effects they impart on their hosts. As many of these organisms are traditionally isolated from food, incorporation in their native form into the host system in many cases is enough to elicit a reaction from host immune cells (e.g., [7,9]). The outcomes noted from *Lactobacillus* culture administration can vary depending both on the type utilized and the ailment being studied. The majority of studies follow a formula where a disease model is generated by a well-characterized agonist and then treated with the *Lactobacillus* culture or derivative. The resulting analysis of cytokine panels and cell surface markers then implicate how the host system responded to the treatment. Different results are desirable depending on the targeted therapeutic avenue. For example, studies on immune system priming aimed at adding protection against pathogen infection would be considered successful with specific T-cell differentiation patterns indicating immune system activation via well-studied Toll-like receptor (TLR) signaling pathways [20]. In this case, increases of mRNA or protein levels of pro-inflammatory cytokines such as IFN-γ, TNF-α, IL-1β, IL-8 or IL-4 would be considered desirable immunostimulatory results. On the other hand, when *Lactobacillus* species are being studied to treat overactive immunity such as with models of colitis characterized by unchecked inflammation events, the specific changes that signify success are of those that attenuate the host immune response such as increases of the anti-inflammatory cytokines TGF-β, IL-10 and IL-22. The establishment of a disease profile that can be reversed or reduced by the addition of the *Lactobacillus* component is therefore promising evidence for use of a specific *Lactobacillus* strain as a living therapeutic.

A wealth of data in recent years has been collected on the influence of *Lactobacillus* cultures on cytokine stimulation in a variety of formats. The introduction of *Lactobacillus* into model systems does not go unnoticed by host immune systems regardless of administration strategy. Considering that cytokines coordinate host responses to different immune events, few exogenous elements can avoid initiating cytokine activity. The innate immune system acts as one of the first lines of defense against pathogen invasion, and its stimulation involves the activation of TLRs [20]. This activation can occur via either epithelial cells or immune cells such as monocytes, and is an important part of a healthy functioning immune system. The activation of immune cells is important for early detection of infections, and therefore such activation can be a desirable therapeutic target. If the appropriate cells are activated to lower the threshold of pathogen detection, a state of immune alertness can be achieved that allows for a faster clearance of the infection. The maturity and differentiation of immune cell types are determinative of the types of cytokines that will be produced, and thus the type of immune response to be elicited. The complexity of T-cell-mediated immunity has been reviewed elsewhere [21], therefore, in light of the focus of this review and for the sake of simplicity in understanding the directions of immunity influenced by *Lactobacillus* species, T-cell differentiation will be crudely lumped into the categories of an active form (T_Helper_1 or T_H_1, T_Helper_2 or T_H_2 and T_Helper_17 or T_H_17) responsible for the secretion of pro-inflammatory cytokines (IL-2, IL-4, IL-5, IL-9, IL-13, IL-25 and IFN-γ), and a modulating form (regulatory T or T_Reg_) responsible for regulation of active T cells via secretion of anti-inflammatory cytokines (TGF-β and IL-10). The role of monocytes in directing immune system activity is also an important factor affected by *Lactobacillus* cultures, specifically dendritic cells (DCs) and macrophages for their TLR signaling tendencies in addition to the T cell differentiating cytokines they produce.

The effect of different *Lactobacilli* species on monocytes and naive T cells has been noted in recent years. Investigators have observed interesting changes in immune cell behaviors in the presence of not only the organisms themselves, but also spent culture media and recombinantly produced cell wall components. For example, macrophages produce decreased amounts of TNF-α, IL-1β and IL-17 in the presence of *L. plantarum* isolates from cocoa fermentation [9]. The same isolate was shown to increase T_H_ cell populations from peripheral blood mononuclear cell (PBMC) cultures in addition to increased amounts of IL-10 from mononuclear cells, indicating the ability of this *Lactobacillus* culture to both attenuate inflammatory signaling through direct monocyte contact and influence T-cell maturation in mixed cultures. These results are not uncommon in this field, as many investigators have noted similar changes in monocyte behavior as well as T-cell maturation. Other examples include the increase in T_H_1 populations in high-fat diet mouse models caused by *L. rhamnosus* and *L. plantarum* [22], the increased T_reg_ populations in enteritis mouse models with *L. casei* [23] and in systemic lupus erythematosus mouse models with *L. paracasei* and *L. reuteri* [24]. Effects on monocytes have also been repeatedly demonstrated such as anti-inflammatory DC maturation in antigen-challenge mouse models by *L. plantarum* [25] and *L. rhamnosus* with IgA [26]. Studies such as these are good examples of the effects of *Lactobacilli* on an organismal scale, wherein the skewing of T-cell populations and DC activity to a host-beneficial profile is detailed more thoroughly in the individual studies.

Investigators attempting to identify more specific molecular mechanisms responsible for the immune cell priming and immunomodulation by *Lactobacilli* cultures found interesting results regarding their adhesion properties with respect to the phagocytotic activity of macrophages. The pilus encoded by the *spaCBA* genes from *L. rhamnosus* strain GG (LGG) was recently found to be integral for direct macrophage contact resulting in anti-inflammatory TLR2 signaling [27]. Studies with human monocyte-derived macrophages similarly showed an increase in T-cell association cell surface markers when incubated with heat-killed *L. casei* cultures [28]. These results were particularly intriguing, for the identification of bacterial invaders by phagocytosis usually initiates internal TLR2 signaling events that pass through NF-κB to ultimately result in upregulation of a large number of inflammation-associated genes that establish a state of emergency throughout the cellular environment. According to these findings, however, not all bacteria are recognized by macrophages to elicit the same TLR signaling fate, as *spaCBA* deletion mutants of the spaCBA pilus did not have the same effect on signaling [27]. Similar results were noted with *L. acidophilus* cultures using DCs, where investigators narrowed the cause down to alternate signaling processes dependent on activation of pathways downstream of TLR2 itself. The data showed that upon uptake of *L. acidophilus*, endosomal degradation as well as downstream activation of pro-inflammatory genes such as IFN-β was prevented [29]. While there is also evidence of *Lactobacilli* inducing both pro-inflammatory [7,30] and seemingly contradicting signaling outcomes [31], the indications of unique interactions between *Lactobacillus* cultures and monocytes [27,29] demonstrate the potential of the immune system to differentiate between pathogenic and probiotic bacteria, which can certainly be exploited by therapeutic strategies against immune system overactivity given the appropriate context and strain usage.

The most established effect of *Lactobacillus* on mammalian cells involves increasing anti-inflammatory activity. While inflammation is an integral part of immunity, there are a number of diseases characterized by unchecked inflammation that damages host tissue [16]. Furthermore, chronic inflammatory diseases can be complex with elusive causes, limiting the number of viable treatment options. For example, mammals contain mechanisms to attenuate inflammation themselves with a number of strategies such as using macrophages and T_Reg_ cells, and some cases of inflammation damage occur when these populations and associated signaling pathways are out of balance from unknown external or auto-induced stimuli. The anti-inflammatory influences of *Lactobacillus* species therefore provide a therapeutic strategy with minimal impact.

The influence of *Lactobacillus* species on immune cell maturation and differentiation discussed above accounts for one facet of their potential therapeutic scope. In addition to their effects on naive lymphocytes, different *Lactobacillus* species can stimulate anti-inflammatory cytokine production in a number of different scenarios (Table 1). The studies summarized in Table 1 involve either animal or cell culture models (or both) and universally report the *Lactobacillus*-induced increases in anti-inflammatory IL-10 and TGF-β as well as reductions of pro-inflammatory TNF-α, IL-1β and IL-6. In contrast to previously discussed T_H_ population enhancements, *Lactobacillus* species in chronic inflammation models tend to reduce T_H_ activity as well as populations that coincide with decreased IL-17, IL-2 and IL-4 (Table 1). The attenuation of T_H_ activity is vital to controlling inflammation, as these pro-inflammatory cells secrete IFN-γ, activate granulocytes and even deactivate T_Reg_ cells. The effects of *Lactobacillus* cultures on mammalian inflammation models seem to be universal, but a few dissenting studies exist that present some confusion. For example, reported increases in IL-10 and IL-6 production from DC isolates that could be interpreted in multiple ways [26]. On one hand, IL-6 can cause anti-inflammatory effects through the inhibition of TNF-α, but on the other hand IL-6 can interact with macrophages in pro-inflammatory events [32–34]. Despite the fantastic data and accessibility that comes with cell culture experiments, this particular example emphasizes their limits, as the larger effects of these two cytokines are unclear. In this case, animal models could provide a vital understanding of the ultimate systemic effects of cytokine production, and would clarify whether the observed increases of both IL-6 and IL-10 either caused or mediated inflammation through the assessment of the induced changes in T-cell populations or the downstream immune activation in surrounding tissues.

**Table 1.** Inflammation mediation studies.

Species	*In vitro*			*In vivo*			Ref.
	Model	Markers		Model	Markers		
		Increased	Decreased		Increased	Decreased	
*L. acidophilus*	RAW264.7	IL-10	TNF-α	Mouse	IL-10		[35]
*L. acidophilus*				Mouse	IL-10	TNF-α, IL-6, IL-1β, IL-17	[36]
*L. acidophilus*				Mouse		IL-17, IL-23, TGF-β	[37]
*L. brevis*				Mouse		IL-1β, TNF-α, TGF-β	[38]
*L. brevis*^†^	RAW264.7		TNF-α	Mouse		TNF-α, IL-1β, IL-6	[39]
*L. bulgaricus*				Mouse, mesenteric lymph nodes	IL-17, TNF-α, IFN-γ, IL-4, IL-2	IFN-γ, IL-6	[40]
*L. bulgaricus*				Mouse, spleen	IL-10, IL-17, TNF-α, IFN-γ, IL-6, IL-4, IL-2		[40]
*L. crispatus*	DC	IL-10					[41]
*L. crispatus*	Mixed lympocytes	IL-10					[41]
*L. delbrueckii*	Bone marrow-derived DC	IL-23					[14]
*L. delbrueckii*	Small intestinal lamina propria	IL-22					[14]
*L. fermentum*	Isolated peritoneal macrophage		IL-6	Mouse		IL-6, IL-17	[42]
*L. fermentum*	PBMC	IL-10	IFN-γ, IL-2, IL-4, IL-13, IL-17				[43]
*L. paracasei*	Differentiated THP-1		TNF-α, IL-1β				[44]
*L. paracasei*	PBMC		IL-6, TNF-α				[44]
*L. pentosus*	Isolated DC	IL-10, TGF-β, IFN-γ					[45]
*L. rhamnosus*	RAW264.7	IL-10	IL-6				[27]
*L. plantarum*	RAW264.7	IL-10		Mouse	IL-10		[46]
*L. plantarum*	RAW264.7		IL-6, TNF-α				[47]
*L. plantarum*	Blood monocyte-derived macrophase	IL-10		Rat	IL-10		[48]
*L. rhamnosus*	Isolated DC	IL-10, TGF-β, IL-6					[26]
*L. plantarum*	PBMC, DC	IL-10	IFN-γ, IL-17, IL-23	Mouse		IL-6, TNF-α	[49]
*L. plantarum*	A549		IL-8, IL-6				[49]
*L. plantarum*				Mouse		TNF-α, IL-6	[50]
*L. plantarum*				Mouse		IL-8, IL-1, TNF-α	[51]
*L. reuteri*				Mouse		IL-6, IL-1β	[52]
*L. reuteri*	Lamina propria lymphocytes, intestinal organoiods	IL-22	TNF-α	Mouse		TNF-α, IL-1β	[53]
*L. reuteri*	Serum			Mouse, serum	IL-2	TNF-α, IFN-γ	[54]
*L. sakei*	Caco-2	IL-10	TNF-α	Mouse	IL-10	IL-17, TNF-α, IL-1β	[8]

† Summary of effects of *Lactobacillus* cultures on cytokine levels in cell culture models (*in vitro*), animal models (*in vivo*), or both. Changes in inflammation markers denoted as increased or decreased based on statistically significant changes from controls as noted in the referenced individual studies. All *Lactobacillus* cultures were living unless denoted with as heat-killed by.

DC: Dendritic cell; PBMC: Peripheral blood mononuclear cell.

Animal studies can be associated with variability and difficulty assigning contributions of specific cell sources, but they are far superior for observing the broad therapeutic outcomes of *Lactobacillus*, such as the effects of downstream signaling on tissue morphology, cell populations and localized responses. Table 1 summarizes a number of inflammation studies with animal models treated by *Lactobacillus* species. As with the primed cell culture studies, controls with induced inflammation by dextran sodium sulfate (DSS) or 2,4,5-trinitrobenzenesulfonic acid (TNBS) are characterized by high levels of pro-inflammatory cytokines (IL-1β, TNF-α, etc.), and upon the administration of *Lactobacillus* there are trends of increased IL-10 and TGF-β as well as decreased pro-inflammatory markers similar to that seen with cell culture experiments. One particular study showed the anti-inflammatory effects described above with *L. sakei* food isolates as comparable with 50 mg/kg of the anticolitic drug sulfasalazine [8]. In this study, *Lactobacillus* cultures reduced TNF-α and IL-1β by 72.6 and 52.9%, respectively, and increased IL-10 to 56.1% in TNBS-induced mouse models. These effects exemplify the potential viability of this anti-inflammatory therapy, as there was no significant difference between *Lactobacillus* and sulfasalazine treatments [8]. As mentioned above, animal models for these types of studies allow broader conclusions to be drawn based on the increased amount of available data. For example, in the study by Chen *et al.* with *L. acidophilus*, decreases in IL-17, IL-23 and TGF-β were noted upon treatment of the inflammation model. Similar to previously mentioned cell culture studies where observed cytokine changes are potentially confusing, this is a case where both pro- and anti-inflammatory cytokines decrease. However, the investigators were able to utilize the wealth of available data to conclude these changes were caused by decreases in T_H_17 populations, and they ultimately represent an anti-inflammatory response. While these results are not typical among recent studies, they represent the clarity that animal studies bring to otherwise complex issues resulting from therapeutic assessments. Similarly, many recent studies have used the wealth of data accumulated from animal studies to conclude anti-inflammatory outcomes from *Lactobacillus* therapies in colitis [36,43,45], enteritis [23], hepatic injury [55] and *K. pneumoniae* infection models [49].

There are a few recent human studies involving the effects of *Lactobacillus* cultures that offer intriguing results. One particular study involved *L. casei* administration to healthy adults, from whom macrophages and T cells were then extracted for *ex vivo* studies [56]. Investigators noted overall lower IL-12, IL-4 and TNF-α levels throughout the 14-week study, and interestingly noted increases in natural killer (NK) cell activation upon discontinuation of dosing. While there was no disease model being studied here, these results still indicate a mediating effect on inflammation, reinforcing the potential of using *Lactobacillus* species for an anti-inflammatory therapy. Adults with metabolic syndrome were administered *L. reuteri* in a separate study, wherein microbiome composition was altered in addition to decreases in IL-6 and soluble vascular adhesion molecule 1 as metabolic syndrome symptom markers [57]. Studies with stressed adults taking *L. plantarum* supplements revealed similar results as in mouse studies with decreases in plasma IFN-γ and TNF-α in addition to increases in IL-10 [58]. The implications of this study, however, are particularly interesting as a relationship is drawn between mental stressors and circulating cortisol and their effects on the immune system, for ultimately this unconventional stimulation of inflammation would otherwise damage host tissue. Therefore, while not affecting perception of stress (albeit physical or mental), the presence of *Lactobacilli* species was shown to enhance immunomodulation and thus prevent subsequent associated tissue damage [59].

### Influence on epithelial cells

The effect of *Lactobacilli* on immune functions is intriguing, especially when considering a therapeutic strategy to combat a systemic inflammatory disorder. Interactions of *Lactobacillus* cultures with epithelial cells comprise another aspect of this therapeutic potential given their role as a physical barrier between lumenal and humoral spaces. As epithelial layers can act as avenues of pathogen invasion in the respiratory, urogenital and gastrointestinal tracts, the influence of *Lactobacillus* cultures on barrier maintenance and their protective effects against pathogen adhesion and invasion are two points of interest for this review.

The gut microbiome has a complex role in its interactions with host nervous and immune systems, but the benefits of this relationship are tethered to the gut itself being a self-contained environment. The intestinal barrier therefore protects the bloodstream from the diverse gut microbiome residing in the lumenal spaces [5]. While these bacteria can contain immunostimulatory lipopolysaccharide and toxins, they are relatively inert when contained within the gut. The breakdown of intestinal barrier caused by the degradation of tight junction proteins (TJP) or epithelial apoptosis can therefore be deleterious to host immune function as a result of these opportunistically pathogenic organisms invading the humoral tissue. As such, intestinal barrier dysfunction has been linked with inflammation in both directions, as the barrier helps to prevent inflammation as a consequence of such gut leakiness, and barrier function and turnover is influenced by some of the same mechanisms that instigate inflammation [60]. In light of this relationship and the previously demonstrated anti-inflammatory influence of *Lactobacillus* species, these bacteria similarly are involved in the maintenance of intestinal barrier integrity.

**Figure 2.**
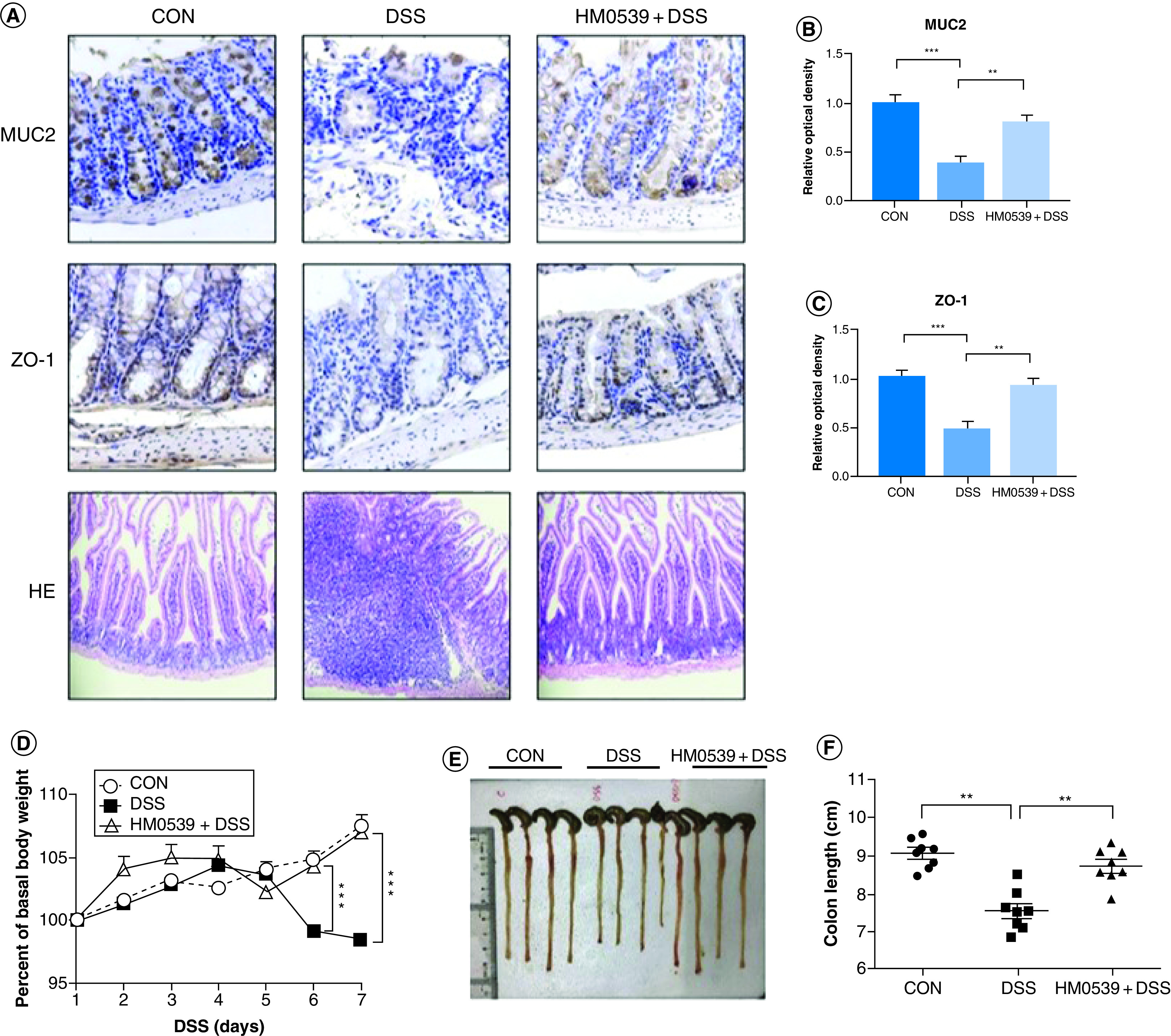
Protective effect of effector molecule HM0539 isolated from *Lactobacillus rhamnosus* GG on dextran sodium sulfate-induced colitis mouse models. Mice were randomly divided into control group (CON), DSS-colitis group (DSS) and HM0539-treated DSS-colitis group (HM0539 + DSS). Mice were gavage fed with pectin/zein control or pectin/zein beads containing HM0539 from days 1 to 9. DSS colitis was induced by adding 3% DSS in the drinking water from days 3 to 9. All mice were sacrificed at day 12, the colitis severity and intestinal dysfunction were evaluated. Immunohistochemical staining and semiquantitative analysis of MUC2 **(A**, upper panel, **B)**, and ZO-1 **(A**, middle panel, **C)** of the colon section. **(A**, lower panel**)** HE staining of colon section **(D)** Body weight of mice from 1 to 7 days after DSS treatment. **(E & F)** Colon length of mice at day 12. Data are given as means ± SEM. **p < 0.01; ***p < 0.001. CON: Control group; DSS: Dextran sodium sulfate. Data taken from [73].

Evidence for the positive effects on intestinal barrier integrity by *Lactobacillus* has existed for decades and is summarized in Table 2. These results originate from cell culture and animal studies in which live cultures, spent media and sometimes isolated surface layer proteins from different *Lactobacillus* species were used to reverse the effects of pathogen- or chemical-induced breakdown of barrier functions. The majority of studies show that *Lactobacillus* treatments maintain trans-epithelial electrical resistance measurements in the face of barrier antagonists as well as decrease pro-inflammatory cytokine concentrations. The effects of *Lactobacillus* treatment on TJP expression are also noted in Table 2, wherein these proteins are generally upregulated as a form of barrier maintenance in the face of the barrier dysfunction inducer. Taken together, these studies indicate that prevention of TJP disruption, trans-epithelial electrical resistance maintenance and promotion of TJP expression in response to stimulated barrier damage are all repeatedly demonstrated effects of a variety of *Lactobacillus* species in both cell culture and animal studies. Figure 2 is an example of intestinal barrier and tissue health assessments in DSS-induced mice with *Lactobacillus* treatment including maintenance of the proteins ZO-1 (a TJP) and MUC2 (involved in mucus layer integrity) via differential staining microscopy (Figure 2A) and the resulting protein abundance measurements (Figure 2B & C). There are a few studies that show *Lactobacillus*-induced downregulation or stabilization of TJPs [61,62], however these studies were carried out differently to assess the effects of *Lactobacillus* species as a prophylactic, and the authors concluded that pretreating cells with *Lactobacillus* cultures prevented the upregulation of TJPs for repair by precluding any initial breakdown of the barrier itself.

**Table 2.** Intestinal barrier integrity studies.

Species	*In vitro*				*In vivo*			Ref.
	Model + challenge	Markers		TEER	Model + challenge	Markers		
		Increased	Decreased			Increased	Decreased	
*L. reuteri*	IPEC-J2 + LPS	Claudin-1, ZO-1	TNF-α, IL-6	Maintained				[63]
*L. plantarum*^†^	IPEC-J2 + ETEC		IL-8, TNF-α, ZO-1, Claudin-1, Occludin	Maintained				[62]
*L. plantarum*	NCM460 + ETEC	ZO-1, Occludin		Maintained				[64]
*L. plantarum* and salmosan	Caco-2 + Salmonella Enteritidis	IL-10, IL-6	TNF-α	Maintained				[65]
*L. acidophilus*	Caco-2 + Salmonella Typhimurium	Claudin^‡^	IL-8	Maintained				[66]
*L. acidophilus* media	Caco-2 + IL-1β		Claudin-1^§^	Maintained				[61]
*L. reuteri*	IPEC-1 + ETEC	IL-10, ZO-1	IL-6, TNF-α					[67]
*L. sakei*	Caco-2 + LPS	Claudin-1, Occludin, ZO-1			Mouse + DSS	Claudin-1, Occludin, ZO-1		[8]
*L. acidophilus* SLP	Caco-2 + TNF-α	ZO-1, Occludin	IL-8					[68]
*L. rhamnosus*					Mouse + ethanol	Claudin-1, Occludin, ZO-1	IL-17	[69]
*L. reuteri*					Weaning piglets + Rotavirus	ZO-1, Occludin		[70]
*L. plantarum*					Mouse + ETEC	Claudin-1, Occludin, ZO-1	IL-1β, IL-6, IL-8	[30]
*L. acidophilus*					Weaning piglets	Occludin	IL-1β, IL-18	[71]
*L. buchneri*					Weaning rabbits	IL-4, ZO-1	TNF-α	[72]
*L. fermentum*	PBMC	IL-10, Claudin-3	IL-2, IL-4, IFN-γ, IL-13, IL-1β		Mouse + DSS	IL-10, Claudin-3	IL-2, IL-4, IFN-γ, IL-13, IL-1β	[43]

† Summary of cell (*in vitro*) or animal models (*in vivo*) studies focusing on intestinal barrier integrity with different species of *Lactobacillus* and associated spent culture media, surface layer proteins or oligosaccharides (in the case of Salmosan). Changes in cytokine (IL-10, IL-6, IL-2, IL-4, IL-13, IL-1β) and TJP (ZO-1, Claudin, Occludin) levels are marked as increasing or decreasing referring to statistically significant changes compared with controls in the referenced works. Results from in TEER are noted as statistically similar comparisons with controls in the referenced works. The notation ‘maintained’ represents experiments with observed statistically significant differences in TEER between controls and pathogen-challenged cultures, but not in cultures exposed to both pathogen-challenge and *Lactobacillus* treatment (e.g., TEER was maintained at control levels). Species used for pretreatment of model.

‡ Quantification of multiple claudin genes.

§ The observed downregulation of TJPs is attributed to prevention of barrier breakdown as discussed in the referenced works.

DSS: Dextran sodium sulfate; ETEC: Enterotoxigenic E. coli; PBMC: Peripheral blood mononuclear cell; TEER: Trans-epithelial electrical resistance; TJP: Tight junction protein.

One particularly interesting study involved the use of *L. plantarum* to condition NKs [64]. Primed NKs were observed to increase anti-inflammatory IL-22 production, and their co-culture with colonic epithelial cells caused increases in ZO-1 and the enzyme Occludin in the face of enterotoxigenic *E. coli* (ETEC) infection. These results act as a reminder of the interplay between multiple cell types as well as the importance and benefit of studying diverse co-culture systems in addition to animal models. As mentioned above, utilizing multiple cell types increases the complexity of the data, but it allows for a broader assessment of therapeutic efficacy.

While the adhesion capabilities of *Lactobacillus* species to host cells can influence immune signaling as described above, it also efficiently blocks pathogen adhesion and subsequent tissue damage. The adhesion characteristics of *Lactobacillus* have recently been studied in a couple of contexts. In one example, the purified adhesin proteins MUB and CmbA from *L. reuteri* were shown to upregulate IFN-γ secretion from T_H_1 cells as well as IL-10, TNF-α, IL-6 and IL-12 from monocyte-derived DCs [74]. Similar results were noted from enhanced interactions of SpaC pilus-containing *L. rhamnosus* GG with epithelial cells [75] and macrophages [27]. In addition to this influence on cytokine stimulation, *Lactobacillus* pretreatment has also been used to prevent pathogen invasion in a number of studies (Table 3). All of the pathogens studied were Gram-negative bacteria except one, and investigators were able to confirm the inhibition of either pathogen adhesion to host cells, invasion due to maintained barrier function, pathogen survival or a combination of each. There is an implication that the adhesion of the *Lactobacillus* species provides a physical barrier that subsequently prevents pathogen adhesion and invasion, but these effects could additionally arise from bacteriocin activity. The *C. albicans* study [76] is interesting in this regard, for it represents the potential for *Lactobacillus* strains to prevent the adhesion and virulence of a eukaryote against which bacteriocins are generally not effective.

**Table 3.** Pathogen adhesion prevention studies.

Species	Cell model	Pathogen	Prevented pathogen activity	Ref.
*L. plantarum*	HT-29	*Salmonella* Typhi	Adhesion	[9]
*L. plantarum*	Caco-2	*Salmonella* Typhimurium	Adhesion, invasion, growth	[6]
*L. plantarum*	Caco-2	*E. coli*	Adhesion, invasion	[77]
*L. plantarum*	Caco-2	*Salmonella enteriditis*	Adhesion, invasion, growth	[78]
*L. reuteri*	IPEC-1	ETEC	Adhesion	[79]
*L. rhamnosus*	Bovine endometrial endothelial	*E. coli*	Invasion	[80]
*L. reuteri*	IPEC-1	ETEC	Adhesion	[67]
*L. rhamnosus*	Bovine mammary epithelial	*E. coli*	Adhesion	[81]
*L. crispatus*	VK2/E6E7 vaginal epithelial	*C. albicans*	Adhesion	[76]
*L. acidophilus*	Caco-2	*Shigella sonnei* and *Vibrio cholerae*	Adhesion, invasion	[82]
*L. plantarum, L. fermentum, L. casei*	Caco-2	*E. coli*	Adhesion	[13]

Summary of epithelial (Caco-2, HT-29, IPEC-1, etc.) and endothelial cell culture studies where *Lactobacillus* species were shown to prevent adhesion, invasion or growth of pathogens including different *Salmonella* and *E. coli* strains.

ETEC: Enterotoxigenic E. coli.

**Figure 3.**
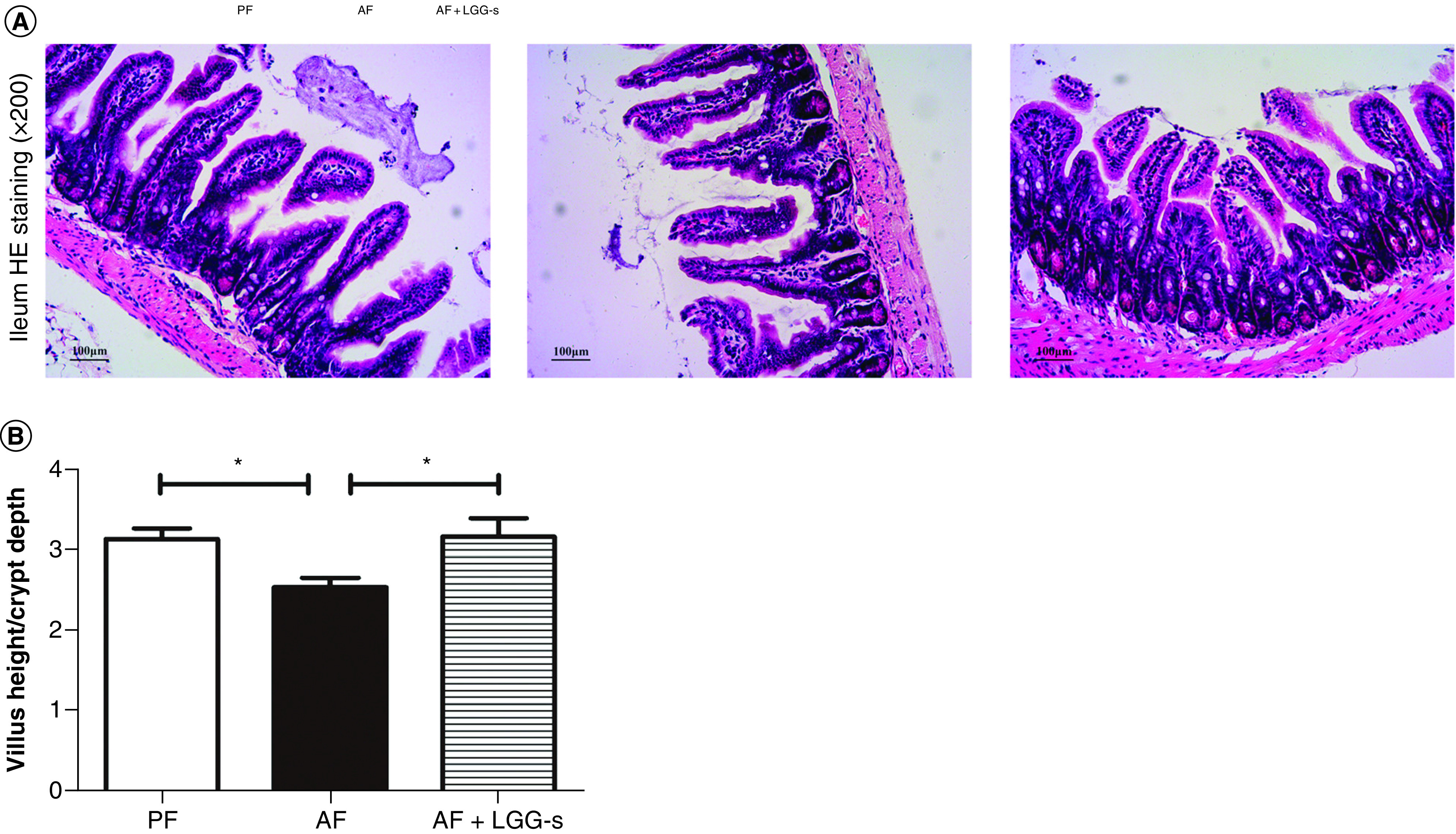
Effect of *L. rhamnosus* GG culture supernatant on villus-crypt junction in ileum. **(A)** Typical micrographs for HE stained ileum sections. Original magnification, ×200. **(B)** Length measurements for villi and crypts from three sections per group were conducted. Ratios of villus length to crypt depth were illustrated as means ± SEM, p < 0.05. AF: Alcohol-fed animals; AF + LGG-s: alcohol-fed animals, treated with LGG supernatant; PF: Control animals. Data taken from [69].

Some of the additional associated benefits to *Lactobacillus* adhesion involve histological protection, including the prevention of colon shortening (e.g., Figure 2E & F), maintenance of villi height/depth ratios for the intestinal lumen (example in Figure 3B) and others resulting in the prevention of weight loss in animal models that might arise due to cellular damage (e.g., Figure 2D). The panels shown in Figure 2 are adapted from a single study and show examples of these protective effects throughout animal model experiments, including the tracking of the organism’s weight (Figure 2D) and colon length (Figure 2E & F). The presence of different *Lactobacillus* species, especially those capable of adhesion, bestows an obvious advantage to the host given the variety of pathogens studied in addition to the immune system priming effects described earlier. As such, *Lactobacillus* species have been shown to relieve host tissue from downstream damage due to apoptosis or inflammation arising from both pathogen infection and disease (Figures 2 & 3). This activity has been noted in animal studies with piglets infected with rotavirus, where barrier function in the jejunal mucosa was maintained by a lack of apoptosis, likely stemming from the inhibition of rotavirus replication by *L. rhamnosus* [70]. The prevention of apoptosis by *Lactobacillus* species was also noted in studies with mice exposed to aflatoxin B1 [83] or *Campylobacter jejuni* [84], Caco-2 cells treated with TNF-α [68] or infected with *Shigella sonnei* and *Vibrio cholerae* [82], and bovine endometrial epithelial cells infected with *E. coli* [80]. These observations are relevant to the prevention of pathogen adhesion, but also to the previously described alteration of TLR activation pathways through their antagonistic effects on NF-κB signaling [8,31,42,44,49,61,62,68,83,85–87]. As a result of helping the host resist direct tissue damage from apoptosis, this activity also improves disease-state symptoms normally monitored in chronic overactive immune disorders such as ulcerative colitis. For the context of ulcerative colitis, these are usually much farther downstream characteristics such as organism weight, colon length, villus height and height/depth ratios that deviate from controls (Figure 2D–F and Figure 3), and animal studies in piglets [70], rats [48] and mice [8,40,42,43,88,89] have all noted such positive effects on these criteria concurrent with *Lactobacillus* treatments. While not directly responsible for these benefits to the host, these recent observations reinforce the therapeutic potential of *Lactobacillus* cultures to supplement host responses to pathogen and disease challenges.

## Delivery of native & recombinant products

**Figure 4.**
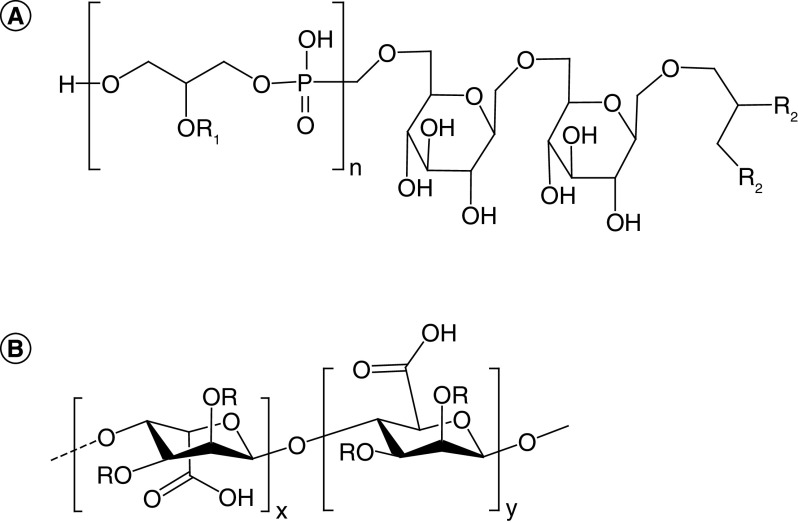
Representative Structures of lipoteichoic acid and exopolysaccharide. **(A)** Lipoteichoic acid base structure contains a repeating glycerophosphate unit (brackets) with substitutions (R_1_ = H, D-alanine or hexose) attached to a glycolipid anchor containing fatty acids (R_2_). **(B)** The exopolysaccharide alginate is shown as an example with repeated glycosidic linkages between hexose moieties containing substitutions (R = H, pyruvate, acetate), where variation between base structures can further arise from the hexose monomers and glycosidic bonds used.

The mechanistic explanations for *Lactobacillus* influence on host immunity present an intriguing mystery. Indeed, they lack the innate immune agonist lipopolysaccharide in their cell wall, but these bacteria also bestow immunological advantages to their hosts as described above. Some of these advantages have been harnessed by investigators to develop promising vaccine administration strategies. One aspect of their influence on host systems is due to natively generated compounds. Some of these compounds have been identified and will be discussed in later sections regarding neural activity. The structural identification of certain bioproducts such as lipoteichoic acids (LTA) and exopolysaccharides (EPS), however, are still under investigation (example base structures of each shown in Figure 4). As these compounds have complex modifications and associated immune effects that vary among bacterial strains, identification of their structural intricacies would provide valuable insight into the molecular mechanisms of their host interactions.

LTA is a surface-bound molecule common to Gram-positive bacteria and has been known to interact with human cells for many years [90]. The Ginsburg (2002) review in particular outlines the role of LTA in inflammation and parallels its activity with that of endotoxin A from Gram-negative bacteria. Interestingly, recent studies have shown LTA from different *L. plantarum* strains to have anti-inflammatory effects in some experimental conditions [86,91,92] while noting upregulation of both pro- and anti-inflammatory cytokines in others [93,94]. Investigators have also noted LTA-induced mucin production in mouse models that ultimately decreased the breakdown of the intestinal barrier and downstream symptoms such as cognitive function [92,95]. Most of the observed activities of LTA are largely dependent upon strain-specific modifications such as acylation and alanylation, but exact structures of the isolated LTAs remain elusive.

EPS is an external bacterial biopolymer of various hexose sugars linked through a number of structure determining strategies (such as α/β-forms of 1,2-, 1,4- and 1,6-glycosidic bonds) that are both strain- and environment-specific [96]. While some base unit structures are known (the structure of alginate is shown as an example in Figure 4B), EPS produced by different bacterial strains can contain substituents and modifications such as acetate and pyruvate that contribute to the structural diversity [97]. This high molecular weight polymer plays roles in adhesion and survival, and in some contexts is an important component of biofilms [98]. While bestowing adhesion capabilities of many *Lactobacillus* species in the gut, EPS has also been shown to add a pseudo-mucus layer to the intestinal barrier capable of preventing pathogen invasion and virulence [79,99–103]. This EPS layer has also been shown to promote anti-inflammatory cytokine production [79,95,99,104–109] and act as a free radical scavenger [110–112], with these activities being enhanced upon chemical modification [111–113]. Investigators have also noted that the addition of EPS to cell culture models can specifically inhibit cancer cell growth, suggesting its potential use as a cancer therapy in addition to the treatment of inflammatory diseases [100,114–116]. The effects of EPS, however, vary with species and strain, and this variation includes increased splenocyte cytotoxicity [117], pro-inflammatory monocyte activity [109,118,119] and the influence of T-cell populations [108,120]. Similar to LTA, however, precise EPS structures are difficult to determine despite recent efforts [79,118,121,122], therefore complicating their efficient widespread therapeutic uses and tethering specific outcomes to specific strains and culturing conditions. Despite the uncertainty associated with these largely uncharacterized native products, their efficient delivery by *Lactobacillus* species has remained an advantage for the use of these organisms as designer chassis for a variety of related purposes.

In fact, *Lactobacillus* species possess a number of characteristics that make them ideal vehicles for biomolecule delivery. In addition to their abilities to influence the immune system described above, these native microbiome constituents can survive the harsh gastrointestinal tract and have subsequently been the focus of engineering efforts [17,123] for the development of vaccine and adjuvant delivery tools. Considering the recombinant techniques developed for these bacteria in recent decades [124,125] in addition to the species variation in gut retention time [126,127], *Lactobacillus* species offer tunability in delivery for the adjustment of therapeutic contact time, exposure and clearance from host systems.

LAB have been used for mucosal delivery of therapeutics for decades. Initial efforts in this field demonstrated methods for *Lactococcus lactis* secretion of bioactive IL-10, effectively modulating inflammatory symptoms in colitis mouse models [128], and subsequent studies have demonstrated similar results in the mucosal delivery [129] and display [130] of IL-10, IL-22 [131], TGF-β1 [132] and serine protease inhibitors [133–135]. Interestingly, surface display techniques can be combined with the potential for some *Lactobacillus* species to prime adaptive immunity [136] in order to create living vaccines.

**Figure 5.**
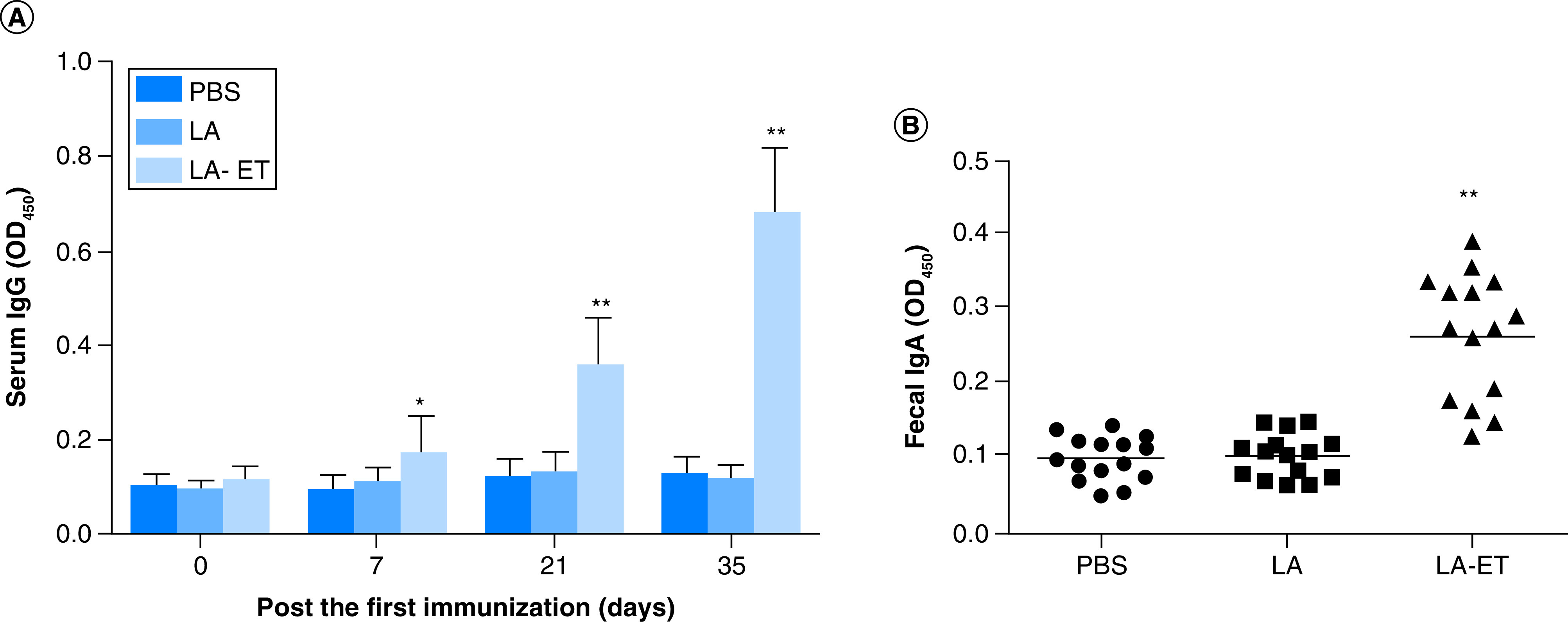
Antibody levels determined by ELISA. The fusion protein espA-Tir-M was used as a coating antigen. Horseradish peroxidase-labeled anti-mouse IgG or IgA was used as the test antibody. **(A)** Serum IgG levels were determined 0, 7, 21 and 35 days after the first immunization. **(B)** sIgA antibody in fecal samples collected on day 35 after the first immunization. Data are shown as the mean ± SD (n = 15) (one-way analysis of variance with least significant difference test). *p < 0.05; **p < 0.001. LA: Wild-type *L. acidophilus*; LA-ET: Recombinant *L. acidophilus* expressing an *E.coli* antigen. Data taken from [140].

**Figure 6.**
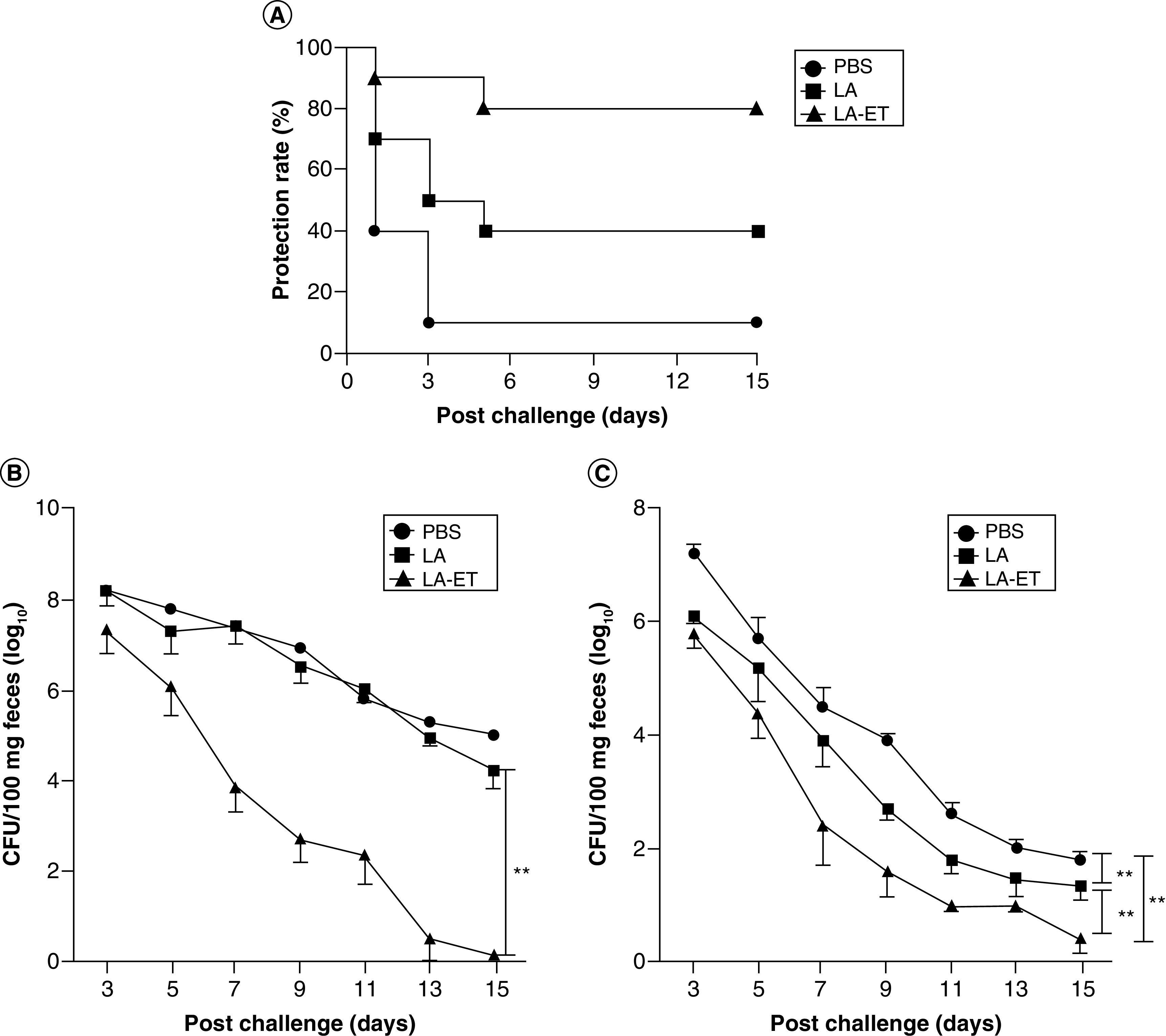
Protection Rates and Fecal Shedding in EHEC O157:H7-challenged mice. **(A)** Protection rates in EHEC O157:H7-challenged mice. The number of mice that died was monitored for 15 days post infection. **(B & C)** Changes in fecal shedding of EHEC O157:H7 in mice. Mice (n = 10) were orally challenged with 10^10^ CFU EHEC O157:H7 either under oral streptomycin treatment condition to clear intestinal flora and enhance EHEC 0157:H7 colonization (n = 10) **(B)** or without streptomycin treatment (n = 5) **(C)** after the last immunization and fecal shedding was monitored for 15 days. The limit of detection for plating was 100 CFU/100 mg feces. Data represent the mean ± SD. Data points in rectangles indicate a significant difference between groups (one-factor repeated measures ANOVA analysis). **p < 0.001. LA: Wild-type *L. acidophilus*; LA-ET: Recombinant *L. acidophilus* expressing an *E.coli* antigen. Data taken from [140].

The mucosal delivery of vaccines is a highly attractive alternative to traditional approaches for a number of reasons. First, as mucosal surfaces are constantly exposed to pathogens and antigens, they play an important role in the activation of both innate and adaptive immune responses, and vaccine delivery in these locations can lead to both systemic and local immunity. As such, the intestine is considered the largest compartment of the immune system [137]. Second, connecting vaccine delivery with gut microflora residency assures optimal administration at the desired locale for immune activation. This particular aspect circumvents common issues of therapeutic delivery such as the necessity for high dosages, complicated formulations, and unique encapsulation strategies to increase the circulation time and efficacy of systemically administered alternatives. Third, *Lactobacillus* species present an ideal balance between immunogenicity and reactogenicity to efficiently achieve immune activation (Figures 5 & 6). By providing both antigen and adjuvant in a single package, *Lactobacillus* vaccines offer the potential to stimulate immunity without the common side effects of other vaccination strategies, such as live attenuated vaccines that can potentially revert to virulent forms in immunocompromised patients or killed pathogens that fail to stimulate a sufficient immune response [125,138,139].

*Lactobacillus* vaccines have been developed in recent years based on the tenets described above and continue to show promise as next generation immunization strategies. Our discussion will include recent conceptual and target highlights as this topic has recently been reviewed [139]. Multiple investigators have shown successful stimulation of immunoglobulin production in response to surface-presented HIV antigens utilizing different co-stimulating factors, such as IL-1β, flagellin and the CCL3 chemokine [141,142], and these strategies have since been extended to different diseases with antigens from tuberculosis [143] and chlamydia [144]. Surface display techniques have also been successful conveying immunity to Influenza H5N1 [145,146], as well as with decoy strategies to combat HIV-1 infection using *L. acidophilus* bearing human CD4 receptors [147]. While traditional display techniques consist of mucin-binding or surface-layer protein fusions, development of newer platforms for surface expression are also currently being optimized such as PilVax [148]. This strategy utilizes an engineered pilus derived from *S. pyogenes* with antigens incorporated into exposed loop regions, is capable of displaying thousands of antigens per cell in *L. lactis*, and has shown potential in recent studies with immunization against *S. aureus* [149].

The potential for *Lactobacillus* vaccines is very promising in terms of the combinatorial possibilities available through strain engineering. Factors such as the *Lactobacillus* species, antigen, surface display or secretion techniques, adjuvant proteins, and immune cell targeting methods can all be varied to achieve the ideal outcome of protection for any given pathogen. In addition to those listed above, successes against various bacteria such as enteropathogenic *E. coli* (Figures 5 & 6) [140,150] as well as diarrheal and respiratory coronaviruses and severe acute respiratory syndrome [83,151–158] have been demonstrated using similar techniques. The development of vaccines for livestock is especially attractive considering the capability to mix cultures into feed rather than employ individual injections to each animal. Furthermore, the scale up process for distribution is also advantageous considering the necessity for only the strain, the vessel and the media, and does not require downstream compound purification or large-scale chemical synthesis. Thus, the use of engineered *Lactobacillus* species for the delivery of beneficial biomolecules or as vaccines is a promising strategy for both mechanistic and practical reasons, and is deserving of further investment of time and resources.

## Production of biogenic compounds to influence host behavior

LAB have been known to generate or influence host concentrations of biogenic compounds for some time. Many of these are decarboxylated amino acids such as tyramine, tryptamine, histamine or γ-aminobutyric acid (GABA) that have various effects on the host nervous system. While histamine has been well studied in the context of immunity for its activity during inflammation when released by mast cells, tryptamine and tyramine (among others) are TAAR1 agonists and GABA is known to act as an integral inhibitory neurotransmitter in the human adult brain. The actions of the affected signaling pathways and their physiological implications have been reviewed recently [159–162], casting an intriguing light on the ability for these compounds to be made by the resident gut microbiota. *Lactobacillus* species are also known to generate short-chain fatty acids (SCFAs) such as acetate, butyrate [163] and propionate [164] in the gut as health-associated metabolic by-products. Other recent reviews have focused on the immunomodulatory capacities [165] and activities in cholesterol homeostasis [166] depending on their presence and abundance in the gut. These SCFAs in addition to the biogenic compounds listed represent a group of natively produced compounds with potential use in a variety of therapies.

Based on previous observations of the abilities for different *Lactobacillus* species to produce SCFAs, investigators have recently examined the effect of different bacterial species on SCFA homeostasis in animal models. Studies have noted alterations in SCFA abundances and ratios in addition to the associated anti-inflammatory benefits with *L. rhamnosus* [167], *L. plantarum* [168,169], *L. johnsonii* [170], *L. sakei* [171]*, L. reuteri* [172] and *L. acidophilus* [173]. Similar results were also seen in human testing with *L. paracasei* [174] and *L. plantarum* [175]. Deeper investigations into these observed effects were carried out in cell culture experiments, wherein positive effects on expression of SCFA cell surface receptors were observed [176,177] despite external stimuli that normally decrease SCFA uptake. Aside from *Lactobacillus* cultures specifically producing SCFAs and influencing host cell surface receptor expression, the actual homeostasis is a complicated dance within the gut that both reflects and sculpts the community structure. Some of the nuances to this system include the effects of antimicrobial peptides (bacteriocins) produced by LAB, capable of killing specific groups of bacteria thereby indirectly altering SCFA ratios [178,179]. Despite the evidence of the health benefits of certain SCFAs, clarifying a universal effect from a single species or even consortium of species has been elusive so far. Optimistically, however, most of the species and strains observed to elicit positive health effects in previous sections have also been shown to positively affect SCFA homeostasis, thereby providing another beneficial facet for their use as a therapeutic strategy.

Administering therapeutics that influence neurotransmitter activity is an intriguing subject, but there are always associated concerns of efficacy, dosage and safety. Considering the complexity of the human nervous system, one question as to the feasibility of bacterial production is whether the generated doses can produce the desired outcome. In the case of GABA, the gut represents a large pool in its generation, consumption, and activity [180], therefore echoing the concerns that, despite being a center for such activity, how much impact can a GABA-producing (or other neurotransmitter) organism actually have on host neural behavior? This topic has been recently reviewed [181] to affirmatively establish the role of the gut microbiota in neurotransmitter production, further promoting the need for the elucidation of physiological effects on humans by specific bacteria.

*Lactobacillus brevis* has been considered a high-capacity GABA-producing organism for over a decade, shown to convert up to 30 mM of L-monosodium glutamate (MSG) to GABA (~3 g/l) in 4 h [182]. While the production of GABA is relatively common feature found in gut microbes [183], this characteristic of *L. brevis* has led to its popularity in animal testing to discern whether the production *in situ* can effect measurable change in neural activity. Recent animal studies have shown that this culture can influence activity in the vagus nerve through serotonin 5-HT_3_ receptors [184], opening the therapeutic scope of *Lactobacillus* administration to different diseases associated with nervous system and hormone signaling. One such disease targeted for study is Type 2 Diabetes, considering the role GABA plays in glucagon release from β cells, and as such investigators have shown *L. brevis* to positively affect glucose reduction in diabetic animal models [185,186]. *In vitro* studies with activated immune cells have also demonstrated immunomodulatory capabilities of these GABA-producing cultures [187].

*Lactobacillus* cultures have also been shown to affect neural activity independent of GABA production. These cases are less determinative without a specific molecule to assign responsibility, but the results are nevertheless intriguing. In studies with both cell culture [188,189] and animal models [190,191], *Lactobacillus* species were shown to cause increases in brain-derived neurotrophic factor that were positively correlated with cognitive activity [190,191]. These particular results emphasize the potential of gut-brain axis research to establish important therapeutic strategies capable of treating highly complex neurological disorders. Such efforts are intriguing considering that an orally administered living therapeutic can potentially provide diverse benefits. Ideally, using the correct consortium of organisms would allow the therapeutic strategy to modulate overactive inflammatory immune signaling, provide protection against enteric pathogens, lower cholesterol as a result of influencing SCFA homeostasis, lower blood glucose levels and provide cognitive recovery in the face of internal and external stressors through neural influencing.

## Conclusion

The previous 5 years of research with *Lactobacillus* have provided increased evidence of their potential as living therapeutic delivery systems. Various species have been shown to influence T-cell differentiation patterns reflecting both immune-priming and immunomodulating pathways in different scenarios. Specific interactions with monocytes have identified signaling pathways initiated by different bacteria to uncover a differential phagocytic end point among bacterial sources, with the immune cells appearing to differentiate between bacterial species. These revelations comprise the foundation of results echoed throughout a wealth of cell culture and animal model studies that overwhelmingly support the role of the *Lactobacillus* species as enhancers of immune regulatory activity. When administered as a pretreatment, investigators were able to take advantage of immune-priming characteristics of some strains to stimulate the immune system and initiate antibody generation, thereby enhancing pathogen protection in a long-term prophylactic way as a living vaccine delivery system. While many of the effects of *Lactobacillus* are caused by factors yet to be elucidated, studies have shown specific culture products such as LTA, EPS, surface layer proteins, SCFAs and GABA to display a variety of effects on both cellular and organismal levels. The potential to deliver a variety of therapeutic options is high with *Lactobacillus*, especially when considering cooperative activity from multiple species. An example of this scope is shown with the improvement of disease symptoms in diabetic models caused by *Lactobacillus* production of GABA in addition to similar strains being used to reduce tissue damage from pathogen-induced inflammation and improve cognitive responses [185–187].

## Future perspective

The optimal understanding of the underlying mechanisms has yet to be reached in order to support the universal deployment of such therapeutics. Many of the tested species have been highly studied with very promising results, but it is hard to imagine these therapeutic strategies as being welcomed by the medical field without further investigations into the identity and molecular mechanisms of species-specific effector molecules to understand and further optimize their therapeutic potential [13]. While some physical products have been identified, the information available is restricted to molecule class alone due to strain-specific complexity and the issues surrounding more determinative identification of modifications such as with EPS and LTA. The lack of information regarding the detailed identification of these effectors is not due to lack of effort, but that efficient and reputable techniques to identify their specific modifications are not readily available. For example, some EPS isolates can have their base structure identified with mass spectrometry after degrading EPS into its monomers with glycolytic enzymes, but characterization of specific substituent modification patterns is more difficult. Substituent determination (identification and position) is possible with nuclear magnetic resonance, but this would need to be done with sufficiently pure monomers and not the intact polymeric isolates, creating difficulty in the elucidation of substituent patterns. Given the importance of EPS structural diversity among strains as it relates to observed immunomodulatory activity, it is imperative to overcome these short-term characterization challenges for these types of therapeutic strategies to continue to gain momentum. An example of the potential advancements of such issues can be seen with the burst of research surrounding GABA with the advent of its discovery as an effector molecule produced by different *Lactobacillus* [192]. The specific identity of different effector molecules in the short-term can then lead to the engineering of designer *Lactobacillus* species to further optimize therapeutic strategies. These longer term goals will be difficult and could require extensive metabolic engineering to produce, given the complexity of strain-specific LTA and EPS modifications.

While a multitude of naturally isolated species have been shown to be effective, there are a number of opportunities available to improve therapeutic efficacy given the identification of necessary mechanisms. As the composition of the gut microbiome has been correlated with therapeutic outcomes, novel antimicrobial peptides generated by recently pioneered machine learning methods [193] could be used to modulate community composition in order to increase the balance of specific SFCA producing strains, or to reduce strains correlated with the onset of neurodegenerative disorders [194]. Furthermore, strain engineering could be carried out to increase the native treatment efficiency, for example using the exogenous expression of the previously tested *L. rhamnosus* GG adhesion protein SpaC to enhance interactions with host cells. While the potential for widespread therapeutic use is great, any incremental step toward these goals requires confirmation from parallel cell culture and animal studies to assess the organismal impact of any treatments. A number of studies reviewed here have done such parallel studies (Table 1), but accessibility and cost make them unfeasible for all research institutions. Furthermore, the transition to voluntary human trials can be similarly inaccessible, reserving this jump for only potential therapeutic strategies with the utmost promise of treatment efficacy. While the route of drug testing is both standardized and difficult for good reason, there is an abundance of available evidence highlighting the benefits of *Lactobacillus* administration for the production and localized delivery of native bioactive compounds.

The successful implementation of *Lactobacillus* species as widely accepted and commonly used therapeutic delivery vehicles is an attainable goal. The tools required for genetic manipulation, cultivation, isolation and testing are already available in most molecular biology labs today. The avenue of vaccine delivery is already a streamlined laboratory workflow whose primary current limitation is the selection of antigens to recombinantly express for the *Lactobacillus*-facilitated exposure to subjects. Without the next generation engineering goals of altering metabolism, adding adhesion properties and generating novel gut community structuring strategies mentioned above, there are still a number of well-studied and readily available strains that have been demonstrated capable of treating various immune and neural-centered abnormalities. The establishment of a next generation of *Lactobacillus* strains would solidify their reputation as therapeutic delivery vehicles with accessible capabilities for scaled-up production and modification. These attributes would allow treatment strategies to quickly evolve as new research emerges regarding the gut microbiome influence on host neural and immune behaviors.

**Executive summary**

- *Lactobacillus* strains have been demonstrated in numerous studies to regulate inflammation, protect the epithelia from pathogen invasion, reinforce the intestinal barrier through the influence of tight junction proteins and stimulate the adaptive immune system.
- Microbial engineering strategies have emerged to modify these bacteria for the production of bioactive compounds to supplement native probiotic activities.
- Further protein engineering strategies have been used to decorate *Lactobacillus* species to display antigens and act as living vaccines that efficiently stimulate specific adaptive immunity through interaction with the gut mucosa.
- Links between different disease progression mechanisms and the *Lactobacillus*-influenced levels of neurotransmitters and short-chain fatty acids highlight the potential for disease modulation as shown in animal models.
- *Lactobacillus* species are emerging synthetic biology platforms that can be paired with machine learning combinatorial methods for bacteriocin production to beneficially sculpt gut microbiome composition for SCFA homeostasis, pathogen control and reinforced immunity.
